# Supplementary material for: The Relationship between the Mediterranean Diet and Vascular Stiffness, Metabolic Syndrome, and Its Components in People over 65 Years of Age
Source: Nutrients. 2024 Oct 12;16(20):3464. doi: 10.3390/nu16203464 (PMC11510127; doi:10.3390/nu16203464)
Supplement: Supplementary file 1 [file nutrients-16-03464-s001.zip › nutrients-3254435-supplementary.pdf]

Table S1. Characteristics of the subjects included with and without MetS in men.

	With MetS (n=362)		Without MetS (n=374)		p value
**Mediterranean Diet**	Mean or N	SD or (%)	Mean or N	SD or (%)	
MD (total score)	5.83	1.82	6.00	2.01	0.233
Adherence to MD, n (%)	135	(37)	153	(41)	0.176
**Conventional risk factors**					
Age, (years)	69.45	3.24	69.49	3.75	0.883
SBP, (mmHg)	140.77	15.61	137.01	18.84	0.003
DBP, (mmHg)	82.41	9.28	79.56	9.84	<0.001
Hypertension, n (%)	308	(85)	240	(64)	<0.001
Antihypertensive drugs, n (%)	235	(65)	175	(47)	<0.001
HDL cholesterol, (mg/dl)	45.88	10.07	55.91	13.22	<0.001
Triglycerides, (mg/dl)	145.19	71.06	97.34	34.54	<0.001
Dyslipidemia, n (%)	268	(74)	296	(79)	0.097
Lipid–lowering drugs. n (%)	135	(37)	105	(28)	0.009
FPG, (mg/dl)	114.58	30.98	94.49	22.03	<0.001
Diabetes mellitus, n (%)	135	(37)	45	(12)	<0.001
Hypoglycaemic drugs, n (%)	121	(78)	34	(22)	<0.001
WC, cm	106.85	9.20	98.47	7.63	<0.001
Obesity, n (%)	156	(43)	50	(13)	<0.001
**Arterial stiffness**					
CAVI	9.52	1.05	9.46	1.05	0.486
baPWV, m sec	15.96	2.53	15.55	2.38	0.025
**MetS and its components**					
Number of components MetS	3.55	0.70	1.58	0.56	<0.001
BP ≥ 130/85 mmHg, n (%)	349	(96)	290	(27)	<0.001
FPG ≥ 100 mg/dL, n (%)	248	(68)	86	(23)	<0.001
TGC ≥150 mg/dL, n (%)	147	(41)	15	(4)	<0.001
HDL-C <40men, <50women mg/dL, n (%)	272	(25)	86	(8)	<0.001
WC ≥88 cm women, ≥102 cm men, n (%)	270	(75)	115	(30)	<0.001

Values are the mean standard deviations for continuous data and the numbers and proportions for categorical data. MetS: Metabolic Syndrome; n: numbers; MD: Mediterranean Diet; SBP: systolic blood pressure; DBP: diastolic blood pressure; HDL-C: high-density lipoprotein cholesterol; FPG: fasting plasma glucose; WC: Waist circumference; BP: blood pressure; TGC: Triglycerides; CAVI: cardio-ankle vascular index; baPWV: brachial-ankle pulse wave velocity.

p value: differences between subjects with and without Mets.

Table S2. Characteristics of the subjects included with and without MetS in women.

	With MetS (n=658)		Without MetS (n=622)		p value
**Mediterranean Diet**	Mean or N	SD or (%)	Mean or N	SD or (%)	
MD (total score)	5.86	1.80	6.40	1.93	0.001
Adherence to MD, n (%)	97	(33)	119	(48)	<0.001
**Conventional risk factors**					
Age, (years)	69.47	3.40	69.72	3.97	0.421
SBP, (mmHg)	137.01	17.07	130.48	19.12	<0.001
DBP, (mmHg)	79.63	10.31	78.33	10.66	0.152
Hypertension, n (%)	254	(86)	145	(59)	<0.001
Antihypertensive drugs, n (%)	212	(77)	117	(47)	<0.001
HDL cholesterol, (mg/dl)	50.12	12.12	68.11	14.99	<0.001
Triglycerides, (mg/dl)	147.57	56.24	91.62	27.96	<0.001
Dyslipidemia, n (%)	266	(90)	212	(85)	0.077
Lipid–lowering drugs. n (%)	133	(45)	90	(40)	0.025
FPG, (mg/dl)	113.87	33.80	86.15	9.52	<0.001
Diabetes mellitus, n (%)	113	(38)	(5)	(2)	<0.001
Hypoglycaemic drugs, n (%)	101	(34)	(5)	(2)	<0.001
WC, cm	99.11	11.17	91.34	10.55	<0.001
Obesity, n (%)	135	(46)	50	(20)	<0.001
**Arterial stiffness**					
CAVI	9.11	1.16	8.94	1.09	0.077
baPWV, m sec	16.39	2.63	15.36	2.64	<0.001
**MetS and its components**					
Number of components MetS	3.60	0.72	1.78	0.42	<0.001
BP ≥ 130/85 mmHg, n (%)	272	(92)	171	(69)	<0.001
FPG ≥ 100 mg/dL, n (%)	197	(67)	9	(4)	<0.001
TGC ≥150 mg/dL, n (%)	123	(42)	4	(2)	<0.001
HDL-C <40men, <50women mg/dL, n (%)	178	(60)	11	(4)	<0.001
WC ≥88 cm women, ≥102 cm men, n (%)	270	(84)	115	(67)	<0.001

Values are the mean standard deviations for continuous data and the number and proportions for categorical data. MetS: Metabolic Syndrome; MD: Mediterranean Diet; SBP: systolic blood pressure; DBP: diastolic blood pressure; HDL-C: high-density lipoprotein cholesterol; FPG: fasting plasma glucose; WC: Waist circumference; BP: blood pressure; TGC: Triglycerides; CAVI: cardio-ankle vascular index; baPWV: brachial-ankle pulse wave velocity.

p value: differences between subjects with and without Mets.
